# Production of Recombinant Alpha Neurotoxin of Scorpion Venom *Mesobuthus eupeus* and Analysis of its Immunogenicity

**DOI:** 10.5812/ircmj.9666

**Published:** 2014-01-05

**Authors:** Ghafar Eskandari, Abbas Jolodar, Masoud Reza Seyfiabad Shapouri, Ardeshir Bahmainmehr, Shahrokh Navidpour

**Affiliations:** 1 Institute of Molecular Biology, National Academy of Sciences of Armenia, Yerevan, Armenia; 2 Department of Basic Sciences, Faculty of Veterinary Medicine, Shahid Chaamran Uiversity of Ahvaz, Ahvaz, IR Iran; 3 Department of Pathobiology, Faculty of Veterinary Medicine, Shahid Chaamran Uiversity of Ahvaz, Ahvaz, IR Iran; 4 Department of Biotechnology-Molecular Genetics, Marvdasht Branch, Islamic Azad University, Marvdasht, IR Iran; 5 Department Veterinary Parasitology, Razi Vaccine and Serum Research Institute, Karaj, IR Iran

**Keywords:** Venoms, Neurotoxin, Scorpion, Recombinant Protein

## Abstract

**Background:**

Scorpion venom is important and rich source of peptides, most of which have been widely used as pharmacological tools for unraveling structure-function relationship of various ion channels. Naturally occurring toxins can be also considered as lead compounds in the development of novel drugs.

**Objectives:**

In this context, the scorpion-derived peptide neurotoxins specific to sodium channels have shown promise as potential therapeutic targets for the treatment of various human diseases.

**Materials and Methods:**

A cDNA library from the extracted RNA was constructed using RT-PCR and semi-nested RT-PCR. DNA sequencing followed by phylogenetic analysis was applied to screen the cDNA library clones. For molecular characterization of the BMK gene we used cloning and recombinant protein expression techniques based on E.coli systems. Then we performed mice immunization and Western blot and Immunodot analyses.

**Results:**

A novel BMK neurotoxin has been cloned, expressed and characterized from the Iranian scorpion *M. eupeus* venom. We analyzed the recombinant BMK by immunoblotting with treated antiserum. The result showed that mice antiserum can react also with scorpion crude venom, so is able to recognize native BMK toxin.

**Conclusion:**

The newly produced recombinant protein BMK revealed to be immunogenic. Moreover, anti-BMK antibodies produced in mice were able to recognize both the recombinant BMK neurotoxin and the one in *M. eupeus* crude venome. Taken together, the molecular characterization and recombinant production of the Iranian scorpion *M. eupeus* venom component can serve as a new probe for further studies of sodium channels function and physiology. This provides a promising perspective for the future design of selective drugs, as well as for research of antivenom production.

## 1. Background

Scorpion venom is a major public health problem in many tropical and subtropical countries, as several species of these animals are highly toxic. This results in significant adult morbidity due to severe systemic envenomation, as well as essential pediatric mortality (1). Furthermore, the scorpion venom represents a naturally occurring vast arsenal (~100,000) of bioactive peptide-toxins. Out of these, only 1% is currently known which have been widely used as pharmacological tools to probe the molecular mechanisms for the functions, biosynthesis, assembly and localization of various ion channels in normal or disease states due to their ability to potently and specifically affect channel functions (2). Naturally occurring neurotoxins can be considered as valuable and useful tools not only for unraveling structure-function relationship of ion channels but also serving as lead compounds in the development of novel drugs. In this context, the scorpion-derived peptide neurotoxins specific to voltage-gated sodium channels have shown promise as potential therapeutic targets for the treatment of various human diseases ranging from pain syndrome, asthma, diabetes, cardiac ischemia and hypertension to chronic inflammation, autoimmune disease and cancer (3, 4). In recent decades, sodium channel scorpion toxins have thus stimulated considerable interest among scientists in pharmaceutical science and neurobiology.

Voltage-gated sodium channels (VGSCs) are large integral membrane proteins, which are critical for the initiation and propagation of action potential in excitable cells. They are composed of a pore-forming α-subunit associated with up to four auxiliary β-subunits (5). To date, seven topologically distinct receptor sites for neurotoxins on the α-subunit of VGSCs have been identified, all of which are linked to specific effects on channel function (6). Venom of Buthidae family scorpions contains a large quantity of long-chain neurotoxins assumed to modulate the sodium channel function; however, detailed data on its venom compounds exist only for a small fraction of it (7). As mentioned above, in tropical countries including Southern parts of Iran and especially Khuzestan province, envenomation by scorpion stings presents a major public health problem due to *Mesobuthus eupeus* of the family Buthidae (8, 9). Antivenom immunotherapy, which still remains the unique specific treatment against scorpion envenomation, is another major area of interest to study buthids toxic components to better understand the envenomation syndrome and improve serotherapy. In recent years, modern molecular biology techniques have provided powerful tools for understanding the complexity of venomous animals. Particularly, the strategy of cDNA library construction followed by molecular cloning and gene expression in heterologous systems has been and still is the most widely employed approach for the characterization of venom toxin precursor-encoding genes (10). Bioengineered scorpion toxins have been monumental to the evolution of channel science, and are now serving as templates for the development of important experimental molecular therapeutics.

## 2. Objectives

The main aim of the work is molecular characterization and recombinant production of a novel α-neurotoxin BMK from the Iranian scorpion *Mesobuthus eupeus* venom, with further assessment of its immunogenicity. The structure-and-function analyses of α-toxin BMK are crucial for the development of new therapeutics, especially with improved immunogenic properties.

## 3. Materials and Methods

### 3.1. Isolation of MeNaTxα-4 Family of α- neurotoxin in Mesobuthus eupeus

The study was performed at the Razi Reference Laboratory of Scorpion Research in Iran. The specimens of scorpions were collected in Khuzestan and transported alive to reference laboratory.RNA was isolated from the venom glands (telson) of twenty scorpions of the species Scorpion Buthida *Mesobuthus eupeus*, Khuzestan (Iran). Semi-nested RT-PCR technique was used to enable the amplification of cDNAs. The first round of PCR was performed using modT-R (5’- cccagatctcgagctcagtg-3’), BMK-F 5- cgcGGATCCTCCGTAAAACGGTTCAAAATG -3’) primers. The second round of PCR was performed using BMK-F and BMK-R 5- cgcAAGCTTACCGCCATTGCATTTTCCT-3’) primers and the PCR products of the initial amplification as templates. The PCR conditions for both rounds were as follows: initial denaturation at 95ºC for 5 min, followed by 35 cycles of denaturation at 94ºC for 40 sec, annealing at 56ºC for 90 sec and extension at 72ºC for 1 min, with a final extension at 72ºC for 10 min. 

### 3.2. Bacterial Strains

The E. coli DH5-α was used routinely for molecular cloning and plasmid transformations and E. coli BL-21 was used for BMK gene expression. Bacteria were grown at 37°C in LB. It was supplemented with 100µg/ml ampicilin where appropriate. The expression vector pMAL-c2x (New England Biolabs) was used to clone the BMK gene sequence for expression in bacteria.

### 3.3. Enzymes and Plasmids

All the restriction enzymes and buffers were purchased from CinnaGen (IR Iran). The enzymes involved were BamHI, HindIII and T4 DNA Ligase. To isolate plasmid from the bacteria E. coli DH5-α, QIAGEN Plasmid Mini Kit [QIAquick Kit = K0513] was employed. The isolation was performed according to the manual. 

### 3.4. DNA Sequencing and Bioinformatics Analysis

Sequence similarity analysis against GenBank database entries was performed using BLAST at the NCBI website (http://www.ncbi.nlm.nih.gov). The theoretical molecular mass for the putative mature peptides and theoretical pI value were estimated by using ProtParam pI/Mw tool program (http://www.expasy.org/tools/protparam). The 3D structure prediction was performed by the Phyre 2 program.

### 3.5. Cloning and Construction of MeNaTxα-4 for Expression in Bacteria

The instruction of competent cells was followed by Sambrook.et al (11) finally; the frozen competent cells were stored at -80°C for long- term usage. The Insert 6 μl of the ligation reaction were used to transfect competent E. coli DH5-α cell. The plasmids of positive colonies were purified by means of the High Pure Plasmid Isolation Kit (QIAGEN). Plasmids were verified by sequencing both sites to confirm the reading frame and to conserve restriction sites. The clone finally obtained was called MeNaTxα-4. E. Coli of the strain Bl-21 were transfected with the corresponding plasmid by transformation heat shock and recovered 30 min a 37°C in LB medium containing 100 µg/ml of ampicillin. In order to confirm expression and to verify the best producing strain of E. coli, a small scale culture of 5 ml each was grown in LB medium and the results analyzed. After the absorbance at OD 600 reached 0.5 units, the cultures were induced with 1 mM IPTG (isopropyl-b-D-thiogalactopyranoside) at 37°C for 3 hours. Cells were harvested and analyzed in SDS-PAGE denaturing gel.

### 3.6. Immunological Assays

The bacteria expressing MBP-BMK recombinant protein was boiled in SDS-PAGE sample buffer and loaded in multiple wells of a 10% polyacrylamide gel. After electrophoresis, the proteins were revealed through immersion of the gel in cold 0.1M KCl and a horizontal strip of the gel containing the recombinant protein was cut to use it for immunization of mice. The gel strip was homogenized in a mortar and resuspended in complete Freund’s adjuvant and mixed by vigorous vortexing at room temperature for 2-3 minutes. Two hundred µl of this mixture containing the recombinant protein was injected intraperitoneally to four female mice. Injections were repeated three time intervals (14, 28 and 42 days) by incomplete Freund’s adjuvant. To determine the production of antibody against BMK protein, the blood was collected 1 week after the last injection by cutting the end of animals’ tails. Sera was separated from the coagulated blood and tested by Western blotting and dot blotting, against the recombinant protein and the crude venom separated from Iranian scorpion *M. eupeus* (obtained from the reference laboratory of the Razi Institute), respectively. 

## 4. Results 

### 4.1. Cloning Full-length Fragments Into Expression pMAL-c2x

**Figure 1.**
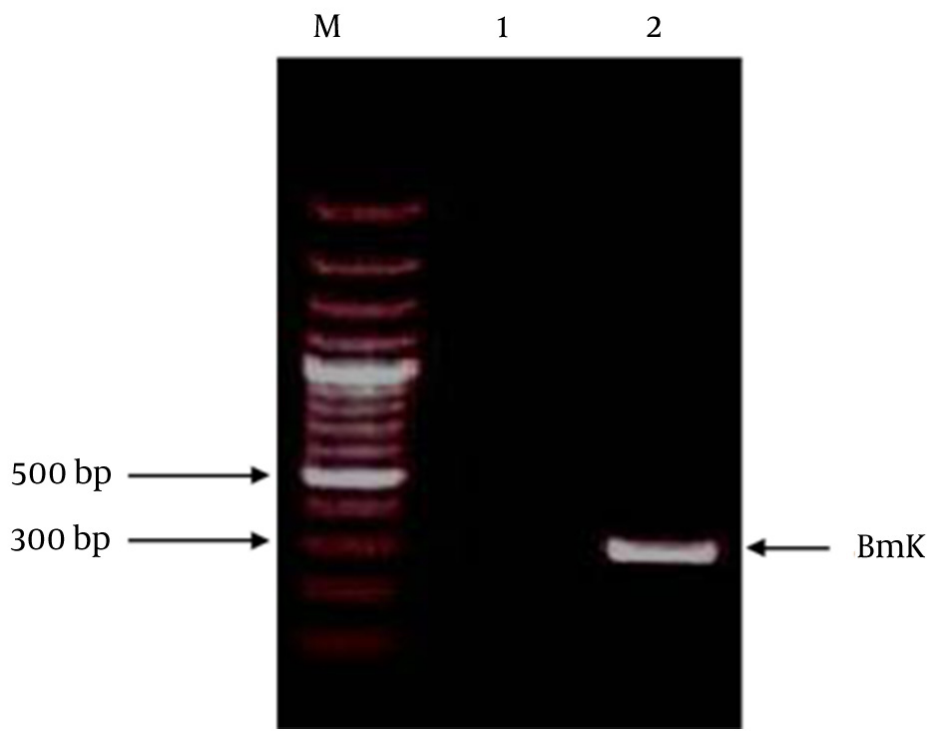
Gel-Electrophoresis Verification of the Amplified BMK Gene Lane M: 100 bp DNA ladder Marker. Lane 1: Negative Control, Lane 2: the BMK Gene.

Amplification of the BMK gene from *M. eupeus* was carried out by RT-PCR using BMK-F and BMK-R primers as described in materials and methods. After the analysis of PCR products, a fragment corresponded to the expected PCR product size 273 bp. The PCR product was purified and used as template for cloning into an expression vector. BMK fragment of α-toxin was digested with BamHI and HindIII. The DNA was subsequently cloned in-frame into BamHI and HindIII sites of expression vector pMAL-c2x. The presence of 273 bp insert DNA fragment was shown after restriction enzyme digestion (Figure 1). 

**Figure 2.**
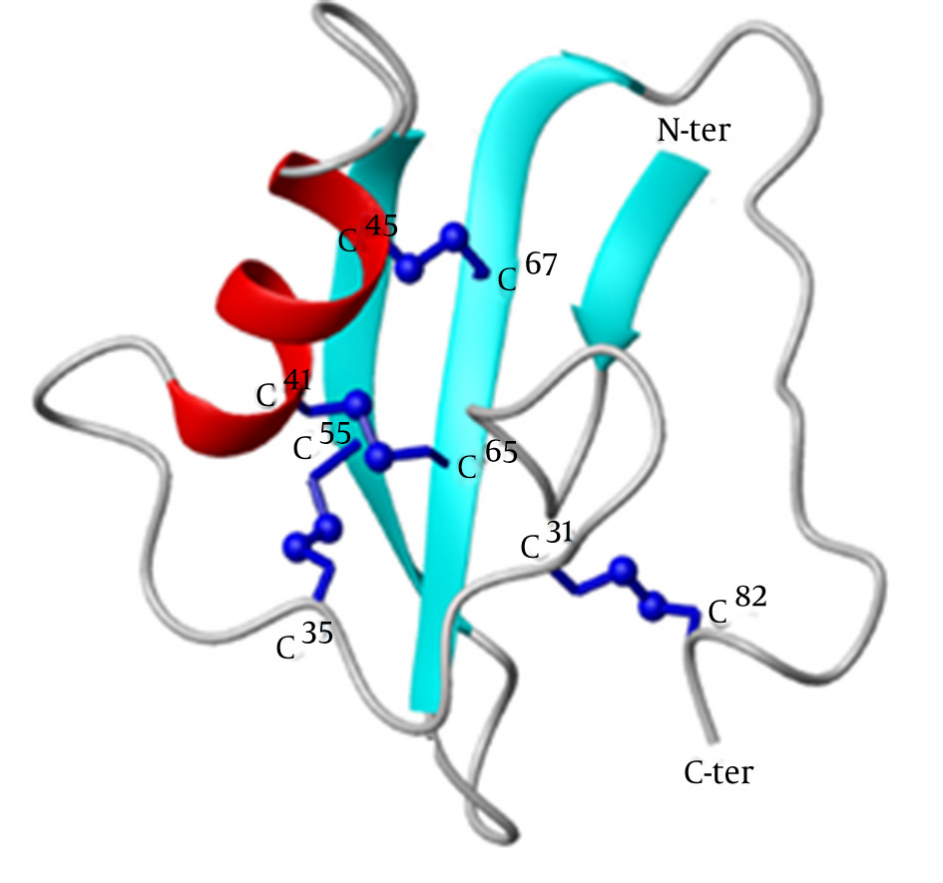
Three Dimensional Model of the MeNaTxα-4 Peptide. The Secondary Structure Elements of the Core and the Four Disulfide Bridges are Shown

Analyzing toxin 3D structures are important because toxin function is related to its structural folding. Inclusion of 3D structural information to toxin sequence analysis facilitates identification of residues that are important for structure and function.According to the molecular model of BMK (MeNaTxα-4), the fold of the polypeptide chain is similar to that of the other long-chain toxins. The protein spatial organization was visualized by its 3D-structure model (Figure 2). 

**Figure 3.**
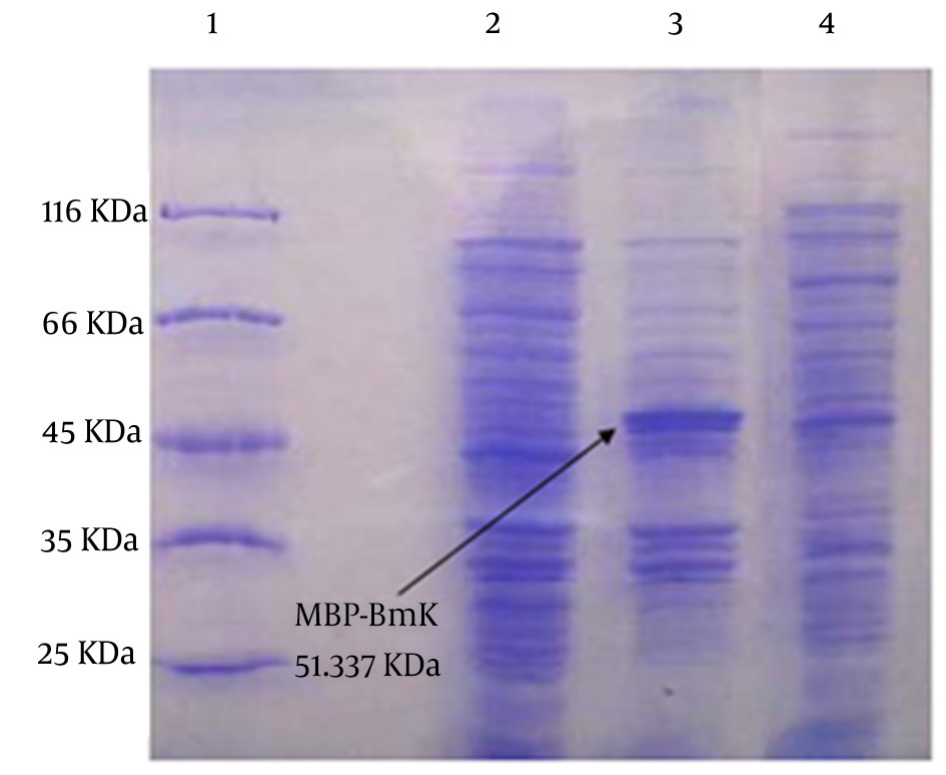
Expression of the Recombinant MBP-BMK Fusion Protein as Detected by Coomassie Blue Stained SDS-PAGE Lane 1: molecular weight marker, Lanes 2 and 3: pMAL-c2x-BMK proteins pattern before and after induction by IPTG, respectively. Lane 4: profile of pMAL-c2x proteins. The protein band corresponding to MBP-BMK is indicated by arrow.

According to the computational model, the peptide is folded into one α-helical conformation and a three-stranded anti-parallel β-sheet. The α-helix is linked to β-strand3 by two disulfide bridges, Cys41-Cys65 and Cys45-Cys67. The loop between β-strand1 and the α-helix is linked to the core of the molecule by a third disulfide bridge, Cys35-Cys55. The fourth disulfide bridge, Cys31-Cys82, links the loop to the C-terminus (Figure 2). It is suggested that the pharmacological versatility displayed by different groups of long chain neurotoxins might have been achieved along evolution via structural reconfiguration of the C-tail. The expression system pMAL-c2x facilitates the production of target protein by fusing it to MBP in the plasmid as detailed in Materials and methods. In our experiment the soluble fraction of MBP-BMK fusion protein was expressed in the *E. coli* BL21 heterologous system and tested by SDS-PAGE (Figure 3). 

The figure clearly shows the presence of an intense protein band with the molecular weight of 51.337 kDa expected for MBP-BMK (9.337 kDa of BMK and 42 kDa of MBP). Thus we concluded that BMK was produced in heterologous E. coli system in soluble and functional form. The results also indicated that this type of expression system is particularly well adapted to disulfide rich membrane proteins.

### 4.2. Immunodot Blot Assay

**Figure 4.**
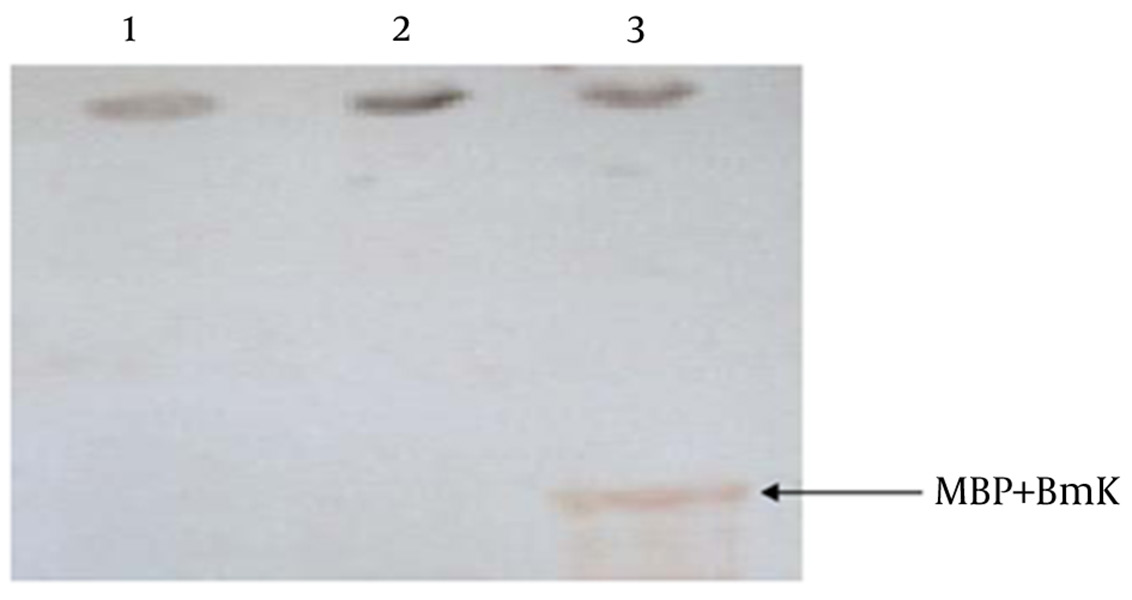
Western Blot Analysis of the Recombinant MBP-BMK Lanes 1 and 2: pMAL-c2x before and after IPTG induction, respectively; Lane 3: recombinant MBP-BMK protein.

Western blot analysis with the anti-scorpion neurotoxin antibodies obtained from mice showed that the expressed recombinant BMK occupied the expected position (Figure 4). In the first two lanes, which corresponded to pMalc2x before and after induction with IPTG, no visible protein bands were observed that could attribute to MBP .Whereas a strict protein band was detected in lane 3 corresponding to the recombinant protein MBP-BMK.

**Figure 5.**
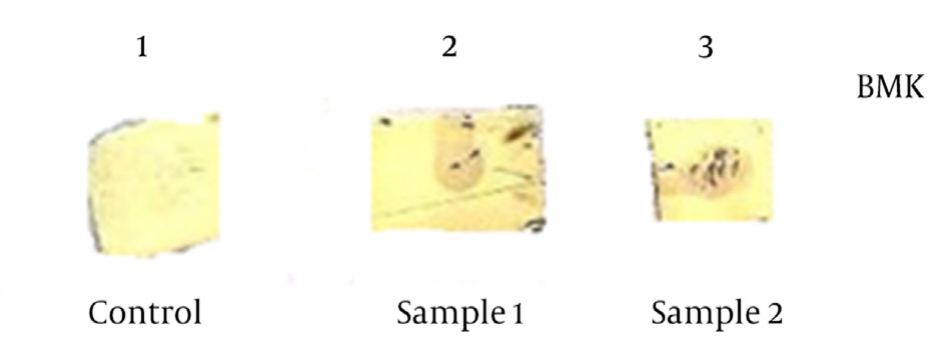
Immunodot Blotting With Antiserum Obtained From Mice and Crude Venom From Scorpion *M. Eupeus* Lane 1: Pure PBS (Negative control), Lanes 2 and 3: positive reactions of antiserum with Crude venom.

It is considered that animal antisera have a number of antibodies reacting with E. coli proteins, which may create pseudo-reactions. However, in our study the reaction is likely related to the antibodies raised against BMK and not MBP, since antibodies elicited against MBP had been removed from the antiserum by treating with E. coli lysate. Therefore, we concluded that the serum obtained from the immunized mice contains antibodies, which are able to recognize the recombinant BMK protein. Thus, the produced recombinant protein was found to be immunogenic. It is clear that handling, transportation and milking of animals alive always poses some risks, especially when dealing with dangerous species for humans, as it is currently done nowadays (12, 13). In order to determine whether the antiserum obtained from mice can react with native BMK toxin from *M. eupeus* crude venom, an immunodot assay was performed (Figure 5). 

The resulted reactions demonstrated that the serum of immunized mice can also react with scorpion crude venom, which corroborated the result that anti-BMK antibodies are able to recognize BMK toxin from the Iranian *M. Eupeus* scorpion. Thus, we produced a recombinant BMK toxin, which was found to be immunogenic, and antibodies produced against recombinant BMK can react with both recombinant and crude venom BMK neurotoxin thereby opening new prospects for the treatment of envenomation. Since this type of peptide contains 4 disulfide bridges, it is expected that the heterologous expression might fold the peptide with possible distinct disulfide arrangements. The variability generated by these various disulfide arrangements should be investigated whether it could present any advantage for the production of neutralizing antibodies.

## 5. Discussion

Following successful construction and planning, unique BMK gene was isolated, expressed and characterized. The results showed that the alpha toxin gene could be cloned into pMAL-c2x and transformed into E. coli DH5-α, and a special E. coli host cells BL21. The bacteria that were used have rare tRNAs as host cell for protein expression. Previous studies have shown that this type of expression system is particularly well adapted to disulfide rich proteins. It has been used to express erabutoxin and BotXIV insect specific toxin of the scorpion *Buthus occitanus tunetanus* (14). Hence, it seems that the entire recombinant product expectedly goes to the inclusion bodies. The result showed that recombinant host cell could express recombinant MBP-BMK protein 51.337 kDa (9.337 kDa BMK and 42 kDa MBP). The fact that this strain produces the recombinant product as inclusion bodies could be due to the experimental conditions used. The protein that was expressed at 37ºC was insoluble, as similarly found in reference (15).Therefore, recombinant BMK gene in pMAL-c2x was transformed into this E. coli strain BL21. After induction, the recombinant E. coli expressed the protein in both soluble and non-soluble fractions. In order to produce higher level of soluble protein, the temperature for bacterial culture was reduced and the result showed that 37ºC was appropriate. The soluble recombinant MBP-BMK was expressed. The protein, which was found to be immunogenic, reacted with antibodies against the recombinant MBP-BMK protein. Immunized mice with the recombinant MBP-BMK protein produced an adequate level of anti-sera titer, which was able to recognize not only the pure toxic peptide but also the soluble venom. The western blot and dot blot analyses with anti-scorpion neurotoxin polyclonal antibodies showed that the expressed BMK protein located in the expected position. Scorpion α-toxins constitute a family of peptide modulators that induce a prolongation of the action potential of excitable cells by inhibiting voltage-gated sodium channel (VGSC) inactivation (16). Therefore, according to the previous studies we also found that the BMK gene is a new member of α-toxin from the Iranian scorpion *M. eupeus*. It belongs to the long chain scorpion Toxin-3 superfamily. Regarding all known toxins in this family, MeNaTxα-4 is sodium channel inhibitor with four conserved disulfide bridges.

These results are quite important, because they open the possibility to scale up the production of the recombinant peptide for immunization purposes using horse (or sheep). This allows production in bigger amounts of anti-venoms against this species of scorpions without the need for field collection or laboratory rearing of specimens for obtaining their crude venoms. The lack of living specimens for venom collection, the high price and small catalog of lyophilized venoms available from providers, and moreover, the several steps of chromatography, represent bottlenecks that limit the available quantities of venom components. Additionally, it is obvious that the handling, transport and milking of animals alive always poses some risks, especially if dealing with dangerous species to humans. This would allow using this material for producing the neutralizing antibodies against this protein from the same species in other countries. Overall, the molecular characterization of the Iranian scorpion *M. eupeus* venom component can be used as a valuable tool to study the structure-function relationship of the toxin with voltage-dependent sodium channels to approach physiological and pharmacological dynamic regulation of sodium-channel targets. This provides a promising perspective for the future design of selective drugs, as well as for research of improved antivenom production.
